# Evolution, Three-Dimensional Model and Localization of Truncated Hemoglobin PttTrHb of Hybrid Aspen

**DOI:** 10.1371/journal.pone.0088573

**Published:** 2014-02-10

**Authors:** Estelle Dumont, Soile Jokipii-Lukkari, Vimal Parkash, Jaana Vuosku, Robin Sundström, Yvonne Nymalm, Suvi Sutela, Katariina Taskinen, Pauli T. Kallio, Tiina A. Salminen, Hely Häggman

**Affiliations:** 1 Department of Biology, University of Oulu, Oulu, Finland; 2 UMR-MD1, Transporteurs Membranaires, Chimiorésistance et Drug-Design, Aix-Marseille Université, Marseille, France; 3 Structural Bioinformatics Laboratory, Department of Biosciences, Åbo Akademi University, Turku, Finland; 4 Institute of Microbiology, ETH-Zürich, Zürich, Switzerland; Emory University, United States of America

## Abstract

Thus far, research on plant hemoglobins (Hbs) has mainly concentrated on symbiotic and non-symbiotic Hbs, and information on truncated Hbs (TrHbs) is scarce. The aim of this study was to examine the origin, structure and localization of the truncated Hb (PttTrHb) of hybrid aspen (*Populus tremula* L. × *tremuloides* Michx.), the model system of tree biology. Additionally, we studied the *PttTrHb* expression in relation to non-symbiotic class1 Hb gene (*PttHb1*) using RNAi-silenced hybrid aspen lines. Both the phylogenetic analysis and the three-dimensional (3D) model of PttTrHb supported the view that plant TrHbs evolved vertically from a bacterial TrHb. The 3D model suggested that PttTrHb adopts a 2-on-2 sandwich of α-helices and has a *Bacillus subtilis* -like ligand-binding pocket in which E11Gln and B10Tyr form hydrogen bonds to a ligand. However, due to differences in tunnel cavity and gate residue (E7Ala), it might not show similar ligand-binding kinetics as in *Bs*-HbO (E7Thr). The immunolocalization showed that PttTrHb protein was present in roots, stems as well as leaves of *in vitro* -grown hybrid aspens. In mature organs, PttTrHb was predominantly found in the vascular bundles and specifically at the site of lateral root formation, overlapping consistently with areas of nitric oxide (NO) production in plants. Furthermore, the NO donor sodium nitroprusside treatment increased the amount of PttTrHb in stems. The observed PttTrHb localization suggests that PttTrHb plays a role in the NO metabolism.

## Introduction

Hemoglobins (Hbs) are ubiquitous in all living organisms. It has been suggested that eukaryotes have acquired all their globins via horizontal gene transfer concomitant with the endosymbiotic events that are responsible for the origin of mitochondria and chloroplast [1]. In the plant kingdom, three kinds of Hbs have been identified: symbiotic (or leghemoglobin), non-symbiotic (divided into groups 1 or 2) and, most recently, truncated Hb (TrHb or GLB3, [2]). Several reviews are available on these proteins [3]–[5]. However, until recently, research on plant Hbs has focused on symbiotic and non-symbiotic Hbs, whereas the number of reports on TrHbs is limited.

TrHbs are widely distributed in eubacteria and plants, and they are also found in some unicellular eukaryotes. Bacterial TrHbs are small hemoproteins with amino acid sequences 20–40 residues shorter than (non)vertebrate Hbs. Because of the deletions throughout the sequence, the three-dimensional (3D) structure of the bacterial TrHb family is a 2-on-2 arrangement of α-helices, compared with the classical 3-on-3 globin fold. On the basis of phylogenetic and amino acid sequences analysis, the family has been divided into three distinct groups: I (HbN), II (HbO) and III (HbP) [6]. The group II TrHbs can be further subdivided into four lineages: Actinobacteria, Proteobacteria, Firmicutes and plants [6], [7]. The 3D structures of *Mycobacterium tuberculosis* HbO (Mt-HbO, [8]) and *Thermobifida fusca* HbO (Tf-HbO, [9]) from Actinobacteria, *Bacillus subtilis* HbO (Bs-HbO, [10]) and *Geobacillus stearothermophilus* HbO (Gs-HbO, [11]) from Firmicutes, and *Agrobacterium tumefaciens* HbO (Atu-HbO, [12]) from Proteobacteria have been characterized. So far, no crystal structure for plant group II TrHb has been solved.

Since the discovery of the *Arabidopsis thaliana* GLB3 (Ath-HbO) [2], several reports on plant *TrHb* expression and localization have been published [13]–[17]. *TrHb* expression has been found in symbiotic structures, and modulation of gene expression has been detected after different abiotic stress treatments [15]–[20]. Hypoxic conditions induce *TrHb* in soybean, grey poplar, cotton, *A. thaliana* and *Prunus* species [15], [18]–[20]. However, in wheat and rice, such effect has not been found [14], [19]. In symbiotic association with nitrogen-fixing bacteria, *TrHb* expression has been detected in the root nodules of *Medicago truncatula*, soybean and *Lotus japonicus* [13], [15], [17]. According to our previous results, the *PttTrHb* expression was enhanced in roots of hybrid aspen (*Populus tremula* L. × *tremuloides* Michx.) dual-cultured with ectomycorrhizal fungi [16].

Based on structural and functional studies, several roles have been proposed for TrHb proteins, including O_2_ transport, nitric oxide (NO) scavenging and detoxification. The 3D structures of group II TrHbs suggested that distal pocket polar residues stabilize the iron-bound ligand through a tight network of hydrogen bonds, which provides the high oxygen affinity [9]–[11], [21], [22]. Ouellet et al. [21] indicated that Mt-HbO might have a role in the redox-mediating signalling while having an implausible role in the uptake and delivery of O_2_. However, Liu et al. [23] reported that Mt-HbO, expressed in *Escherichia coli,* might be an oxygen collector and/or reservoir that maintains cell respiration under hypoxic conditions. In plants, Ath-HbO had a moderate O_2_ affinity, indicating its potential role as O_2_ transporter [2]–[3].

As bacterial TrHbs are known to detoxify NO [24], Vieweg and co-authors [13] proposed that, similarly, the MtTrHb1 and MtTrHb2 proteins of *M. truncatula* (Mtr-HbO) could suppress NO accumulation during nodule and mycorrhizal symbioses. In our previous paper, we suggested that in hybrid aspen, PttTrHb may be involved in the modulation of NO levels in early reactions involved in root growth, in response to symbiosis with the ectomycorrhizal fungi [16]. In an *A. thaliana* mutant, the lack of the Ath-HbO protein inhibited the germination of seeds at elevated temperatures where the NO lifetime was increased and the germination restored with the addition of an NO scavenger, which suggests a potential role for TrHb as an NO scavenger [25]. In wheat, TatrHb was shown to interact with chloroplast proteins PSK-I and PsbS1, indicating a function involved in photosynthesis [26]. *TatrHb* transcript levels have been shown to increase in roots and leaves treated with NO-releasing compound sodium nitroprusside (SNP), indicating a potential role of TatrHb in NO scavenging or detoxification. Recently, Hemschemeier et al. [27] revealed that in order to survive in hypoxic conditions, unicellular green alga *Chlamydomonas reinhardtii* requires the participation of group I truncated hemoglobin, THB8, in NO-dependent signaling pathway.

Previously, we have shown that hybrid aspen possesses two Hbs, PttHb1 and PttTrHb, and that PttHb1 is able to function *in vivo* as an NO scavenger with an applicable reductase [16], [28]. The aim of the current study is to deepen the knowledge concerning PttTrHb by focusing on the protein 3D structure, phylogeny, localization and expression. In order to reveal the functionally important residues in the hybrid aspen TrHb, we made a structure-based multiple-sequence alignment of bacterial and plant HbO sequences, including PttTrHb. Based on sequence alignment, a phylogenetic analysis is also presented here. As very little is known about plant HbOs at the structural level, we have constructed a homology model of PttTrHb and present a comparison of the heme-binding site with the known TrHb structures and sequences. Furthermore, we have immunolocalized PttTrHb in *in vitro* -grown hybrid aspens and studied the *PttTrHb* expression in relation to *PttHb1* using RNAi-silenced hybrid aspen lines.

## Materials and Methods

### Plant material, growth conditions and SNP treatment

The hybrid aspen (*Populus tremula* L. × *tremuloides* Michx.) lines V613 and V617 used in the experiments were originally produced as described in Häggman et al. [29]. *In vitro* shoots of the lines were proliferated on modified semi-solid MS medium (full strength of C_10_H_12_FeN_2_NaO_8_; half strength of other micro and macro nutrients; 2.22 µM BA and 2.85 µM IAA; sucrose 30 g L^−1^; [30]) under 16∶8 h light/dark photoperiod (140–150 µmol m^−2^s^−1^) at 24°C. To induce root formation, shoots were transferred onto semi-solid growth-regulator-free MS medium containing sucrose 15 g L^−1^.

The V613 plants were grown either three or eight weeks on rooting medium, after which their adventitious and lateral roots, leaves and stems were collected. Leaves 0.5–1 cm in length at the top of the plants and leaves 2–3 cm in length at the middle of the plants represented young and mature leaves, respectively. In order to examine the effect of NO on the accumulation of PttTrHb, plant roots were incubated in water containing 200 µM SNP in a hermetically closed box for 5 h before cutting.

### Sequence alignment and modelling studies

**Figure 1:**
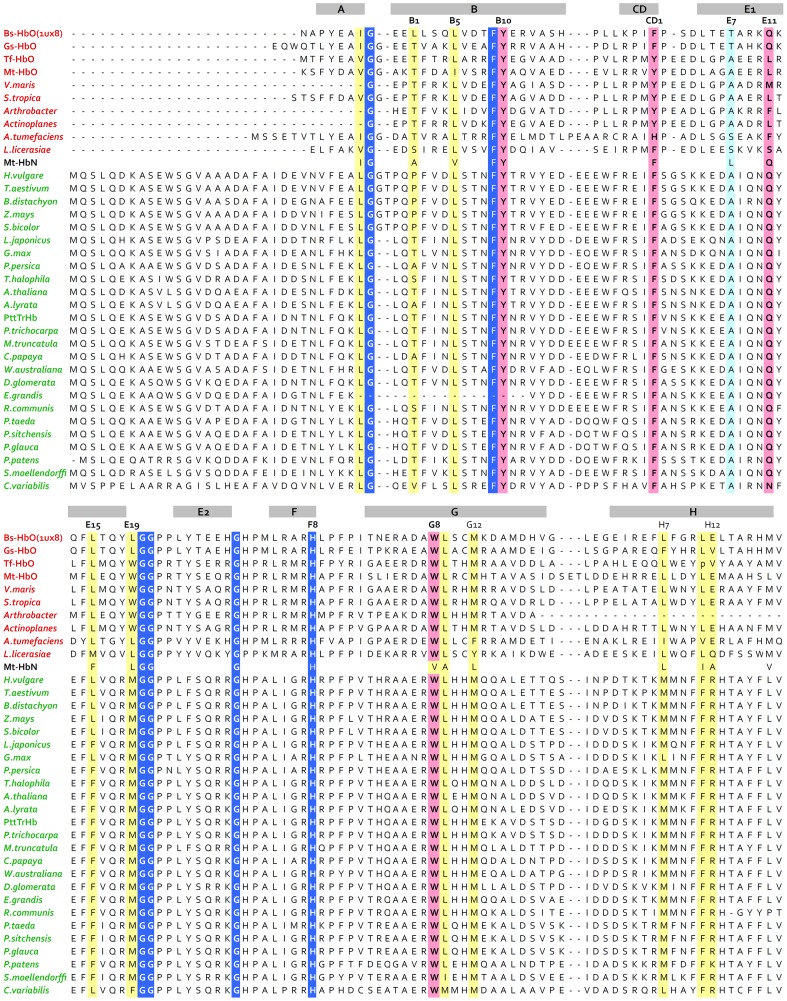
Structure-based sequence alignment of selected group II TrHb sequences from bacteria and plant species. The sequence alignment was generated by superposing the structures and then aligning other bacterial and plant sequences. The α-helices of the X-ray structure of Bs-HbO (1UX8) are shown as grey background on the top of the alignment. The α-helices in vertebrate Hbs with 3-on-3 arrangement of α-helices are conventionally labelled as A, B, C, D, E, F, G and H, with topological sites numbered sequentially within each α-helix [70]. The same labelling of α-helices and topological sites are used here for the truncated Hbs. The conserved Gly motifs and F8His residues are in blue background with white font. The distal site residues B10, CD1, E11 and G8 are in pink background. All residues in the protein matrix tunnel cavity, originally in Mt-HbN (also added to the alignment), are in yellow background. The distal cavity residues discussed in the text are in pink background. The gate residue E7 is in cyan background.

Using BLAST (blast.ncbi.nlm.nih.gov), protein sequences and structures similar to PttTrHb were searched from the nonredundant protein sequence database and protein structure database (Protein Data Bank, PDB), respectively. Twenty-five plant sequences (as presented in Fig. 1) with 66–83% sequence identity to PttTrHb were selected. The BLAST search against PDB revealed five bacterial structures: *B. subtilis* (1UX8; [10]), *M. tuberculosis* (1NGK; [8]), *G. stearothermophilus* (2BKM; [11]), *T. fusca* (2BMM; [9]) and *A. tumefaciens* (2XYK; [1]), which all have less than 33% sequence identity to PttTrHb. To aid sequence alignment, five more bacterial HbO sequences from *Verrucosispora maris, Salinispora tropica, Arthrobacter* sp. FB24, *Actinoplanes* sp. SE50/110 and *Leptospira licerasiae* were selected from the BLAST search against the non-redundant protein sequence database. To identify structurally conserved regions, we superimposed the structures using the program VERTAA in the Bodil suite [31], and pairwise sequence alignments were then combined to generate a structure-based sequence alignment. Other bacterial and plant sequences together with PttTrHb were aligned to the structure-based sequence alignment. The sequence comparisons were done using the program MALIGN [32]–[33]. Based on the multiple sequence alignment, the structural model of PttTrHb was constructed with MODELLER [34] using the default parameters and refinement procedure. The crystal structure of Bs-HbO (1UX8; [10]) was used as the structural template. The PttTrHb model with the lowest value of the objective function derived by MODELLER was visualized in Bodil, and it shows the best heme group coordination. The model was validated with the Swiss Institute of Bioinformatics QMEAN server for model quality estimation [35]. The QMEAN score (0.67) and the Z-score value (−0.74) for the model were well in the allowed region of the density plot for the QMEAN scores of the reference set experimental structures.

### Phylogenetic analyses

For studying the evolution of plant TrHbs, the phylogenetic analyses were performed using maximum likelihood, neighbour-joining and maximum parsimony algorithms [36]. The amino acid sequences for the analyses were obtained by BLAST searches against the PttTrHb sequence in NCBI databases (http://www.ncbi.nlm.nih.gov). Additionally, the TrHb sequences of peach (*Prunus persica* L.) and rose gum (*Eucalyptus grandis* W. Hill ex Maiden) were taken from the Phytozome database (http://www.phytozome.net). Phylogenetic analyses were conducted in MEGA 5.10 (build 5121019). The bootstrap method [37] with 500 replicates was used to evaluate the confidence of the reconstructed trees. Bootstrap values between 70% and 100% have been suggested to indicate significant support for a branch [38].

### Production of recombinant hybrid aspen PttTrHb protein

The *PttTrHb* gene was previously identified by Jokipii et al. [16]. Recombinant PttTrHb protein was produced in *E. coli* BL21 codon plus (Stratagene) using the pET expression system (Stratagene). For the production of the recombinant protein, *E*. *coli* cells were grown in 50 mL Luria-Bertani medium, supplemented with chloramphenicol (34 mg mL^−1^) and carbenicillin (50 mg mL^−1^), at 37°C and 250 rpm until the OD_600_ was higher than 0.6. Expression of the *PttTrHb* gene was induced by the addition of IPTG (Isopropyl β-D-1-thiogalactopyranoside) with a final concentration of 1 mM. Induced cells were cultivated for 2 h, chilled on ice for 10 min, centrifuged (4°C, 5000 g, 5 min) to pellet the bacteria, and the supernatant was removed. The pellet was resuspended in a pH 8-solution containing 50 mM NaH_2_PO_4_, 0.5 M NaCl and 1 mg mL^−1^ lysozyme and incubated on ice for 30 min. Cells were broken by sonication (MSE Soniprep 150, Sanyo GallenKamp PLC, Leicester, U.K). The supernatant was recovered after centrifugation (4°C, 3000 g, 15 min). Recombinant 6×His tagged protein was purified from the lysate by affinity chromatography through a nickel-charged resin column (Ni-NTA agarose, Qiagen). Elution of recombinant protein was effected with a pH 8-solution containing 45 mM NaH_2_PO_4_, 0.45 M NaCl and 0.25 M imidazole.

### Protein extraction, SDS-PAGE and immunoblotting

Soluble proteins from leaves and stem were extracted according to Karppinen et al. [39] from 100 mg of fresh powder with a 50 mM sodium borate buffer pH 9 containing 12% (w/v) polyvinylpolypyrrolidone (PVPP), 10 mM dithiothreitol (DTT) and 50 mM ascorbic acid. Proteins were quantified spectrophotometrically by the Bradford method [40] using bovine serum albumin (BSA) as a standard. Two hundred and fifty micrograms of proteins were loaded in sodium dodecyl sulfate-polyacrylamide gel electrophoresis (SDS-PAGE) gels with a final acrylamide concentration of 12% (w/v) in separating gels and 3% (w/v) in stacking gels. After migration in a Mini-Protean II electrophoresis system (Bio-Rad) at 4°C at 200 V, the proteins were transferred on a polyvinylidene difluoride (PVDF) membrane (Bio-Rad) during 1 h 45 min at 100 V at 4°C using a Mini Trans-Blot Electrophoretic Transfer Cell (Bio-Rad).

The protein membrane was blocked overnight at 4°C with 6% (w/v) dry non-fat milk in TBST [20 mM TrisHCl (pH 7.5), 500 mM NaCl, 0.3% (v/v) Tween 20]. The membrane was incubated 1 h 15 min at room temperature (RT) with an affinity-purified rabbit polyclonal antibody (GenScript) raised against the PttTrHb specific peptide NFYNRVYDDEEEWF. The specificity of the affinity-purified PttTrHb antibody was confirmed by the Western blot technique (Figure S1). After washes with TBST and 30 min in blocking buffer, the alkaline-phosphatase-conjugated anti-rabbit secondary antibody (Bio-Rad) was applied at a 1∶3000 dilution for 1 h at RT under shaking. Before the detection process, washes of the membrane with TBST were done and the substrate solution [100 mM TrisHCl (pH 9), 100 mM NaCl, 5 mM MgCl_2_ and NBT/BCIP (nitroblue tetrazolium chloride/5-bromo-4-chloro-3-indolylphosphate)] was applied in the dark at RT according to the manufacturer's instructions (Roche Molecular Biochemicals). The recombinant PttTrHb protein obtained in *E. coli* was used as a positive control. Blots were digitalized with a VersaDoc™ imaging system (Bio-Rad) driven by Quantity One 1-D analysis software (Bio-Rad).

### Immunolocalization of PttTrHb

Plant materials (roots, stems and leaves) were fixed in 4% (w/v) paraformaldehyde in 100 mM sodium phosphate buffer (pH 7.4, 23 mM NaH_2_PO_4_, 61 mM Na_2_HPO_4_, 1.5 M NaCl) overnight under shaking and dehydrated in a graded ethanol series. Fixed material was embedded in paraffin (Merck) after infiltration with *Tert*-Butanol. Sections of 10 µm were cut with a Microm HM325 microtome and mounted on SuperFrostPlus slides (ThermoScientific) and fixed by drying overnight at 40°C [46].

The paraffin was removed with Histochoice® clearing agent (Amresco) and sections were rehydrated in a graded ethanol series. Slides were put in 10 mM sodium citrate buffer (pH 6) for 30 min at 95°C for the heat-induced epitope retrieval and cooled down 45 min. Sections were incubated for 10 min in PBS (137 mM NaCl, 2.7 mM KCl, 10 mM Na_2_HPO_4_, 1.8 mM KH_2_PO_4_, pH 7.4) and 30 min in a blocking buffer (5% BSA in PBS). The primary affinity-purified antibody against PttTrHb was diluted at 1∶3000 in the blocking buffer and sections were incubated overnight at 4°C with 400 µL of this solution. As controls, the omission of the primary antibody and the preabsorption of the primary antibody (with the purified PttTrHb and an incubation of 5 h at RT under shaking before use) were done. Sections were washed three times for 10 min in PBS, incubated 5 min in the blocking buffer and alkaline-phosphatase-conjugated secondary antibody (Bio-Rad) was applied at a 1∶3000 dilution for 1 h at RT. After four washes of 5 min with PBS and incubation for 5 min into 100 mM TrisHCl (pH 9) containing 100 mM NaCl, sections were incubated for 2 h in the dark at 25°C in the same solution with 5 mM MgCl_2_ and NBT/BCIP according to the manufacturer's instructions (Roche Molecular Biochemicals). Slides were mounted with Euparal (Roth) and sections were photographed under light microscope (Nikon Optiphot-2).

### Silencing of hybrid aspen *Hb* genes

In order to generate RNAi lines, 201 and 203 bp fragments of the *PttTrHb* gene were PCR amplified with the primer pairs 5′-TATGGATCCATTGGACGCCATCGACCATT-3′ (forward) and 5′-TATATCGATATGCTTACAAGGAACACGCC-3′ (reverse), and 5′-TTACTCGAGATTGGACGCCATCGACCATT-3′ (forward) and 5′-TACGGTACCCCATGCTTACAAGGAACACG-3′ (reverse) from hybrid aspen cDNA, respectively. The former pair introduces BamHI and ClaI, and the latter XhoI and KpnI digestion sites (underlined) to the ends of the specific DNA fragment. BamHI-ClaI and XhoI-KpnI double digested fragments were cloned into pHANNIBAL (CSIRO, Clayton South, Australia) hairpin RNAi vector in antisense and sense orientation, respectively. For silencing construct of PttHb1, two 201 bp cDNA fragments were amplified with the primer pairs 5′-ATAGGATCCCCGTGCAGCTAAGGAAAG-3′ (forward) and 5′-TATATCGATGCTGATCATAAGCATCTC-3′ (reverse), and 5′-ATACTCGAGCCGTGCAGCTAAGGAAAG-3′ (forward) and 5′-CAGGGTACCGCTGATCATAAGCATCTC-3′ (reverse). The resultant PCR products were cloned to pHANNIBAL as described above. Subsequently, the sequence verified *Hb* RNAi constructs were released with NotI digestion from the plasmids and inserted into pART27 cloning vector [41].

The *A. tumefaciens*-mediated genetic transformation of hybrid aspen line V617 was performed using the strain C58C1 pGV3850 [42], [43] according to Häggman and co-workers [29] with the following exceptions. The wounded leaf and shoot pieces were pre-cultured on the callus-production medium (0.5 µM BA and 4 µM 2,4-D) for 2 days. For co-cultivation, overnight culture was diluted to OD_600_ 1±0.2 with liquid growth-regulator-free MS medium.

### Quantitative RT-PCR

RNA was extracted from 30 mg of hybrid aspen shoots using the automated magnetic-based KingFisher™ mL method (Thermo Electron Corporation) with MagExtractor® Total RNA Purification Kit (TOYOBO) according to the manufacturer's instructions. Five independent RNA extractions were performed per each hybrid aspen line. Total RNA yields were measured with OD_260_ analysis using a NanoDrop ND1000 spectrophotometer (NanoDrop Technologies) before treating the RNA samples with DNase I (Thermo Scientific) for 20 min at 37°C for the elimination of genomic DNA. The cDNA synthesis was conducted with SuperScript III reverse transcriptase (Invitrogen) using 1 µg of total RNA. The expression of *PttTrHb* (EF180084), *PttHb1* (EF180083) and alpha-tubulin (*TUA*; AY229882) was determined by using the primers presented in Jokipii et al. [16]. In addition, ubiquitin-conjugating enzyme 2 (*UBC2*) with the forward 5′-GGGATGGAGGGACGTTTAAG-3′ and reverse 5′-GGGTTTGGATCACAGAGCA-3′ primers was used in the real-time RT-PCR. The real-time RT-PCR reactions of 20 µL consisted of 1 x LightCycler® 480 SYBR Green I Master (Roche Applied Science, Penzberg, Germany), 0.5 µM of each primer and 2 µL of 1/10 diluted cDNA sample. The reactions were run as duplicates using a LightCycler® 480 (Roche Applied Science) with a PCR program starting with 10 min incubation at 95°C followed by 45 cycles: 10 s at 95°C, 10 s at 60°C and 10 s at 72°C. The melting curve analysis of the LightCycler® 480 software was used to confirm the specificity of the primers. The relative expression of *PttTrHb* and *PttHb1* were calculated using the positive calibrator normalized procedure with two reference genes, *TUA* and *UBC2*. With both reference genes the calculated relative expression values were comparable (data not shown) and, therefore, only the results obtained with *TUA* are presented. The differences between the control line V617 and the RNAi lines were statistically examined either with the two-sample t-test or the Wilcoxon rank-sum test using the R software package 2.5.1 [44] with the graphical user interface, R Commander [45].

## Results

### 3D model of PttTrHb and evolution of plant TrHb

In order to examine the possible ligand-binding residues in plant TrHbs, we constructed a homology model of PttTrHb. Firstly, a sequence alignment was constructed using ten group II bacterial TrHb sequences, which included five structures (PDB codes: 1UX8, 2BKM, 2BMM, 2XYK, 1NGK; see M & M for details). To evaluate sequence conservation and to assist the comparative modelling of PttTrHb, an extensive multiple sequence alignment was then performed by adding 25 plant sequences (Fig. 1). Of the possible template structures, Bs-HbO showed the highest sequence identity, 33%, with PttTrHb. Therefore, the cyanide-bound X-ray structure of Bs*-*HbO was chosen as a template to construct the 3D model of PttTrHb. B9Phe, B10Tyr, G8Trp, F8His and three glycine motifs are well conserved both in bacterial and plant HbO sequences (Fig. 1). The plant TrHb sequences clearly share higher sequence identity with each other than with the bacterial sequences.

**Figure 2:**
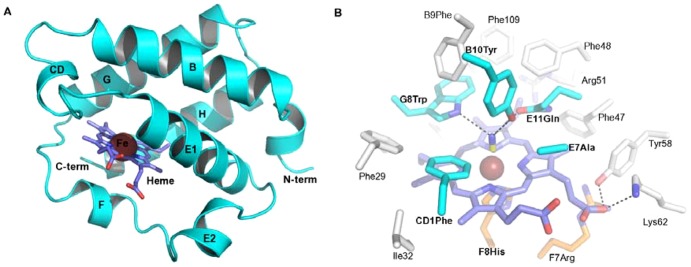
Homology model of PttTrHb and heme pocket residues. A: Cartoon representation of the PttTrHb model in cyan colour. The heme is shown as sticks coloured as C, slate; N, blue; O, red and Fe, orange. B: Heme pocket residues shown in sticks. The proximal residues F8His and F7Arg are in C, orange. The carbon atom in heme is shown as slate. The cyanide ligand is with a yellow carbon. Same conventional vertebrate Hb labels are used for the ligand binding residues in cyan (see Fig. 1). Other residues (in white) are numbered by residue numbers. In the PttTrHb model, same residue numbering is used as 1UX8.

The 3D model of PttTrHb adopts a 2-on-2 sandwich of α-helices that is characteristic of the TrHb family (Fig. 2A). It lacks 24 and 20 residues from the N- and the C-terminus, respectively. The distal heme cavity (B9Phe, B10Tyr, CD1Phe, E7Ala, E11Gln and G8Trp) of TrHbs is highly conserved within plant sequences (Fig. 1). Of these residues, all except E7Ala (E7Thr in Bs-HbO and Gs-HbO) are also conserved in Bs-HbO and Gs-HbO. On the distal site of the heme, E11Gln is hydrogen bonded to the cyanide ligand (Fig. 2B). E11Gln is conserved in plants except for *C. variabilis* HbO, where it is conservatively replaced by asparagine. B10Tyr is involved in the hydrogen-bonding network with the ligand, whereas E7Ala, CD1Phe and G8Trp provide hydrophobic environment for the ligand. The conserved G8Trp, characteristic of group II and III TrHbs, forms a hydrogen bond to the cyanide ligand (Fig. 2B). These results suggest that PttTrHb has a heme distal ligand-binding pocket similar to Bs-HbO and Gs-HbO.

In the previous studies on the group I TrHbs, a protein matrix tunnel/cavity system connecting the protein surface to the heme distal pocket has been suggested to support ligand diffusion to/from the heme distal pocket [47]–[50]. Contrary to Mt*-*HbN, the 3D structures of Mt*-*HbO [8] and Bs-HbO [10] display a restricted protein matrix tunnel due to increased volume of side chains at the topological positions (B1Leu, B5Leu, G8Trp, G9Leu, G12Met, and H12Glu in Bs-HbO), which partly fill the protein matrix tunnel space. In HbOs, most of the tunnel cavity space is filled by the conserved G8Trp. Like Bs*-*HbO, PttTrHb has identical residues at these positions in the tunnel cavity, except for H12Glu that corresponds to a surface arginine residue in PttTrHb. At other positions in the tunnel cavity in PttTrHb, there are slightly bigger residues: E15Leu^Bs-HbO^→ Phe^PttTrHb^, E19Leu^Bs-HbO^→Met^PttTrHb^, H7Leu^Bs*-*HbO^→Met^PttTrHb^ and H11Ile^Bs*-*HbO^→Phe^PttTrHb^ (Fig. 1). Thus, the protein matrix tunnel is clearly further restricted in PttTrHb.

Contrary to the highly conserved E7Gln/Leu in HbNs, the ligand seems to be anchored by a small distal site residue (Ala/Thr/Ser) at the position E7 in HbOs. Of different HbOs, plants have a conserved apolar alanine at E7 compared with polar residue (Ser/Thr) in bacterial HbOs (Fig. 1) and, therefore, both have little hindrance at the entrance to the heme distal cavity. These differences at the gate residue position might affect the accessibility of diatomic ligands to the heme distal site and support the role of E7 as a gate residue in plants and bacteria. In conclusion, the tunnel cavity in PttTrHb and studied plant TrHbs are identical to Ath*-*HbO but different from Bs-HbO, Gs-HbO and Mt-HbO.

**Figure 3:**
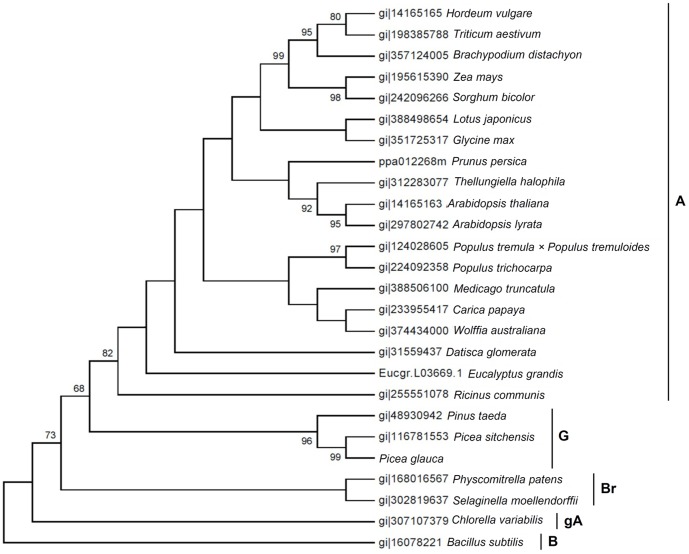
Phylogenetic analysis of plant truncated hemoglobin (TrHb) proteins with significant bootstrap values. A: angiosperms, B: bacteria, Br: bryophytes, G: gymnosperms, gA: green alga.

In the phylogenetic analysis of plant HbO and the Bs-HbO, the maximum likelihood (ML) tree (Fig. 3), as well as the neighbour-joining and the maximum parsimony trees (data not shown), gave consistent results. The TrHb-sequence-based phylogeny was in accordance with the current view on the evolution of green plants in which morphologically simple photosynthetic forms, such as unicellular green algae, gave rise to multicellular forms. Furthermore, morphologically simple plants, such as bryophytes, were followed by more complex flowering forms with highly developed breeding mechanisms at the top of plant phylogeny [51]. In the phylogenetic tree, the main branches for bryophytes, gymnosperms and angiosperms were separated with high bootstrap supports (72%, 68% and 82%, respectively). Thus, the results were consistent with the view that plant TrHbs originated from a horizontal gene transfer event from an ancestor of present day *Chloroflexi* to either the ancestor of all eukaryotes or the ancestor shared by algae and land plants [1].

### Relative expression of *PttTrHb* and *PttHb1*

The association between the two *Hb* genes, *PttTrHb* and *PttHb1*, was examined by producing RNAi lines in order to silence the expression of the *Hb* genes. According to the relative expression calculated with *TUA* as a reference gene, the *PttHb1* expression in the PttHb1*-*RNAi line H3 was 26% of the expression of the control line V617, and the difference between the lines was significant based on the pairwise comparison (*p*<0.05; Table 1). By contrast, in the H3 line, the expression of *PttTrHb* was two-fold higher compared with the control line, suggesting that the decrease in the production of *PttHb1* increased the expression of *PttTrHb* (*p = *0.095). In the PttTrHb*-*RNAi line T3, the *PttTrHb* expression was only 25% of the expression of the control line (*p*<0.05), whereas the T2 line showed expression of 61% of the expression level determined for the control line (*p* = 0.092). The *PttHb1* expression in the PttTrHb*-*RNAi lines did not differ from the control line (both *p* values >0.1) but nonetheless showed consistent trend with the expression pattern of *PttTrHb* in the T2 and T3 lines (Table 1).

**Table 1:** Expression of *PttTrHb* and *PttHb1* in control line V617 and RNAi-silenced lines, H3, T2 and T3.

Line	Type	*PttTrHb*	*PttHb1*
V617	control	0.99±0.17	0.94±0.23
H3	PttHb1 silenced	2.13±0.71	0.26±0.09
T2	PttTrHb silenced	0.61±0.11	1.46±0.19
T3	PttTrHb silenced	0.25±0.08	0.82±0.22

Means ± SE (n = 5) calculated using *TUA* as reference gene.

### Localization of PttTrHb protein in hybrid aspen tissues

**Figure 4:**
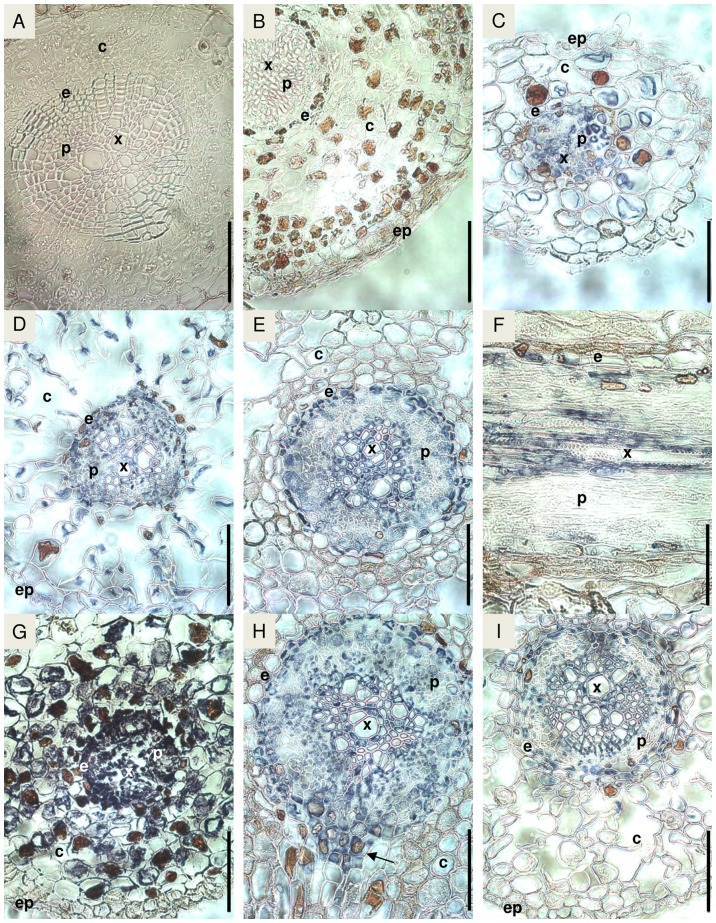
Immunolocalization of *PttTrHb* protein in hybrid aspen roots. Negative controls were obtained by root section incubated in A, without primary antibody or in B, with primary antibody preabsorbed with the recombinant PttTrHb. C to H: plant grown in normal conditions. C, Transversal section (TS) of lateral root. D, TS of adventitious root of plant rooted for 3 weeks. E, TS of adventitious root of plant rooted for 8 weeks. F, Longitudinal section of adventitious root of plant rooted for 8 weeks. G, TS of adventitious root tip of plant rooted for 8 weeks. H, TS of adventitious root of plant rooted for 8 weeks at the point of emergence of a lateral root (arrow). I, TS of adventitious root of plant rooted for 8 weeks after 5 h of SNP-treatment. c: cortex, e: endodermis, ep: epidermis, p: phloem, x: xylem. Bar  =  100 µm.

In the lateral root sections of plants grown in normal conditions, PttTrHb protein was present in the vascular cylinder, endodermis and cortex (Fig. 4C). The same localization pattern was also observed in the three-week-old adventitious roots (Fig. 4D). In the eight-week-old adventitious roots, PttTrHb was only detected in the vascular cylinder (especially in xylem) and the endodermis (Fig. 4E, F). At the apex of adventitious roots, the protein was also present in the cortex area (Fig. 4G). PttTrHb was also specifically expressed at the site of lateral root initiation (Fig. 4H). The SNP treatment of 5 h was not found to affect the localization of PttTrHb in the adventitious (Fig. 4I) or lateral roots (data not shown).

**Figure 5:**
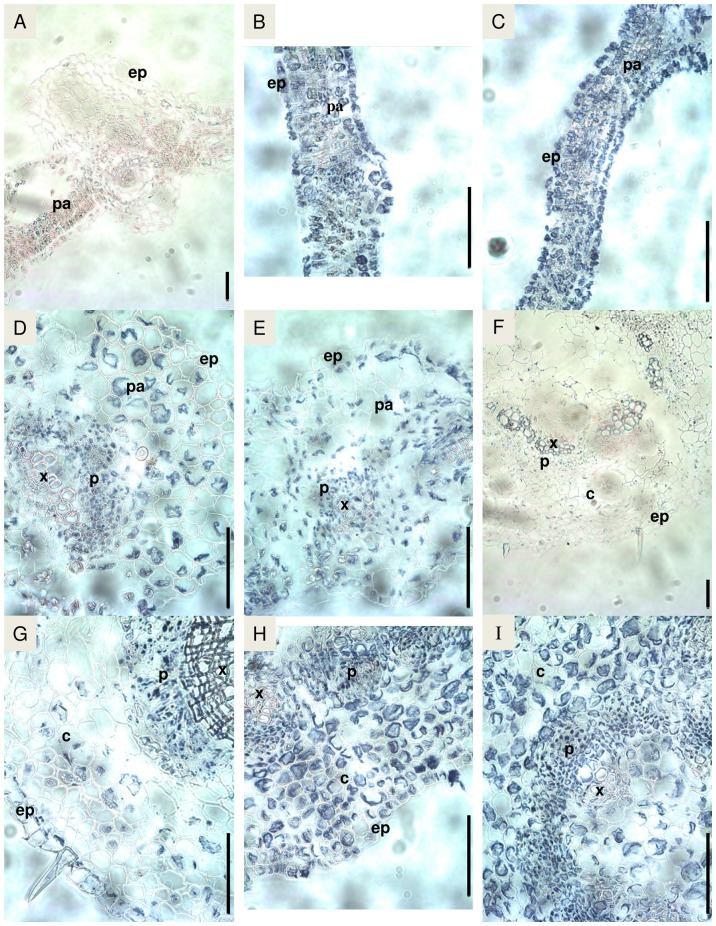
Immunolocalization of PttTrHb protein in hybrid aspen leaves and stem. A, Negative control obtained with leaf section incubated with primary antibody preabsorbed with the recombinant PttTrHb. B, Transversal section (TS) of young leaf from plant grown in normal conditions. C, TS of young leaf from 5 h SNP-treated plant. D, TS of mature leaf from plant grown in normal conditions. E, TS of mature leaf from 5 h SNP-treated plant. F, Negative control obtained with stem section incubated with primary antibody preabsorbed with the recombinant PttTrHb. G, TS of stem from plant grown in normal conditions. H and I, TS of stem from 5 h SNP-treated plant. c: cortex, ep: epidermis, p: phloem, pa: parenchyma, x: xylem. Bar  =  100 µm.

When leaves of different ages were compared, PttTrHb was detected in vascular tissues, in parenchyma cells, and in epidermis cells in young leaves of plants grown either in normal conditions (Fig. 5B) or under NO stress generated by SNP (Fig. 5C). By contrast, the expression was mainly abundant in vascular tissues (especially in phloem) of the mature leaves in aspen grown in normal conditions (Fig. 5D) and SNP-treated hybrid aspens (Fig. 5E). In the stem tissues, PttTrHb was found in the pith, the vascular tissues (xylem and phloem), cortex and epidermis (Fig. 5G). After 5 h SNP treatment, PttTrHb was found abundantly in all cells of the section (Fig. 5H, I). The analysis also revealed that the amount of PttTrHb increased specifically in cortex and epidermis compared with the plants grown in normal conditions (Fig. 5G).

## Discussion

The function of plant TrHbs has been connected to various cellular processes that are linked to NO-dependent signaling pathways [2], [13], [15]–[20], [27]. However, the role of TrHb has been shown to be less essential than that of the non-symbiotic Hbs in the modulation of NO levels [28], [52]–[53]. In the present study, we show the modeled structure and distribution of hybrid aspen TrHb. The results support the view that PttTrHb is potentially involved in the NO metabolism with a putative role in O_2_ transport. Since there was no structural information available for any of the plant TrHbs, we constructed a homology model of PttTrHb that folds as a 2-on-2 sandwich of α-helices characteristic of the TrHb family. The structural data from group II TrHbs, including characterized TrHbs from Actinobacteria, Firmicutes and Proteobacteria [8]–[12], suggest that the distal pocket polar residues stabilize the iron-bound ligand through a tight network of hydrogen bonds, which provides the high oxygen affinity. However, large variations in the affinity for ligands (O_2_, CO, NO and cyanide) has been observed between microbial TrHbs [2], [9]–[11], [21], [22]. The different ligand-binding properties could result from varying ligand-binding residues at the distal site (B10, CD1, E11 and G8) and differences in the tunnel cavity residues. The distal residues for PttTrHb, *A. thaliana* and other plant HbOs are almost identical to Bs- and Gs-HbO. However, Ath-HbO, which has identical distal site to PttTrHb and other plant TrHbs, shows different kinetics and lowest affinity for O_2_ compared with Mt-, Tf-, Bs- and Gs*-*HbO [2], [12]. This could be due to the apolar E7Ala in Ath-HbO (conserved in plant sequences), instead of the polar E7Thr in Bs- and Gs-HbOs, [10] and to the revealed differences in the matrix tunnel cavity system of plant HbOs compared with bacterial HbOs. Since the distal site and tunnel cavity residues are identical in Ath*-*HbO and PttTrHb, the binding properties and kinetics of PttTrHb are likely to be similar to those of Ath-HbO. This suggests that PttTrHb could also display a moderate O_2_-binding affinity and might be a potential O_2_ transporter. However, further studies are needed to verify the role of the residue at position E7.

The non-symbiotic Hb1 proteins and mRNA have mostly been found in the vascular tissues of plants [17], [54]–[55]. The distribution of PttTrHb predominantly found in the vascular tissues of roots, stems and mature leaves in the present study shows consistency with the localization of NO. Production of NO has been found in the xylem and phloem of *Zinnia elegans* stem [56], in xylem and epidermal cells of pea (*Pisum sativum*) seedlings [57], and the phloem of *Vicia faba* leaves [58]. In roots, Guo et al. [59] have detected NO around the root apex region of tomato. Furthermore, NO has been reported to play a central role in lateral root development in tomato as the five-day-lasting SNP application induced the formation of lateral roots [60]. In the present study, PttTrHb protein was specifically observed at the area of lateral root formation. In eight-week-old adventitious roots, the expression of PttTrHb was strongest in the xylem, pericycle and endodermis, and, in root apex, PttTrHb protein was also found in both the vascular tissues and the cortex. Differences in the localization of non-symbiotic *Hb2* (SOLly *GLB2*) mRNA have also been reported as the *Hb2* transcripts were more abundant in young than in mature leaves of tomato [61]. Moreover, NO localization has also been shown to vary depending on the age of plant tissues [62]. Corpas et al. [62] localized NO in pea leaves and showed that the endogenous NO production decreased with the leaf age and in senescent leaves, and that NO was only present in vascular tissue. Similar developmental-stage-dependent variation was also observed in the PttTrHb localization, i.e. in the young leaves and roots, the distribution of PttTrHb was more extensive than in mature tissues.

Because of the consistency between the detected distribution of PttTrHb and NO production in plants [56]–[59], [62] and our previous studies [28], we examined the effect of SNP on the accumulation of PttTrHb. The 5 h SNP treatment was found to increase the amount of PttTrHb in cortex and epidermis of hybrid aspen stems (Fig. 5): this is in accordance with our earlier results which showed that the SNP treatment induced the expression of the *PttTrHb* gene [28]. Similarly, Wang et al. [26] reported that the transcript levels of *TatrHb* can be increased by using SNP treatment [26]. Interestingly, it was also found that TatrHb interacted with photosystem I and II subunits, PSK-I and PsbS1, respectively [26], the latter having a central role in pH- and xanthophyll-dependent nonphotochemical quenching (NPQ) [63]. Previously, it has been shown that NPQ declines under stress conditions in the leaves of *A. thaliana* lacking Ath*-*HbO [64]. However, by using cPTIO, a specific scavenger of NO, the authors found that NPQ can be preserved. Thus, the observed differences in the PttTrHb distribution between young and mature leaves, in the present study, may be connected to the putative senescence associated processes including NPQ [65] and NO [62] decline.

We have previously shown that PttTrHb alone does not rescue an NO-sensitive yeast mutant during NO treatment [28]. Rogstam et al. [66] have also shown that mutations of the *yjbIH* operon containing YjbI (Bs-HbO) and YjbH (cytosolic protein of unknown function) induced a hypersensitivity to SNP in *B. subtilis*. The insertion of the *Bs-HbO* gene alone was not able to restore the survival level of *yjbIH* mutants exposed to SNP. The authors concluded that YjbI and YjbH act together and hypothesized a role for these proteins in nitrosative stress management. Consistently, it is possible that a protein co-operating with TrHb also exists in plants or as recently demonstrated for the first time with *C. reinhardtii* cells [27], TrHb could act as a mediator of NO-dependent signaling pathway. In the present study, the reduced *PttHb1* expression in the PttHb1 silenced line resulted in enhancement of *PttTrHb* expression. The observed expression patterns may indicate changed NO level in Hb1 RNAi plants compared to non-transformed controls since NO concentration is known to have an inverse relationship to the level of Hb1 expression in plants [67]–[69]. The expressions of *PttHb1* and *PttTrHb* in the RNAi silenced lines suggest that the functions of the two genes are connected.

## Conclusion

The current study gives new insights into the 3D structure, phylogeny and localization of PttTrHb. The multiple sequence alignment and the structural model of PttTrHb suggest that PttTrHb and other plant TrHbs have a Bs*-*HbO- and Gs-HbO-like ligand-binding pocket. Due to an Ala in E7 position (Thr in Bs- and Gs-HbO) and even more restricted tunnel cavity, PttTrHb might not, however, show similar ligand-binding kinetics as Bs-HbO and Gs-HbO. Instead, the identical distal site and the tunnel cavity residues in Ath*-*HbO and PttTrHb indicate that PttTrHb might be a potential O_2_ transporter like Ath-HbO. The phylogenetic analysis corroborated the link between plant and bacteria HbOs by indicating the vertical evolution of the plant TrHb proteins from a bacterial TrHb. Moreover, in this study, for the first time, the localization of PttTrHb protein was shown in leaves, stem and roots of hybrid aspen. In mature hybrid aspen organs, PttTrHb was predominantly present in the vascular system. PttTrHb was also detected at the area of lateral root formation, and a difference in the localization of PttTrHb was observed between young and mature leaves and roots. The effect of the NO-donor (SNP) was found in stem tissue after 5 h of incubation. The amount of PttTrHb increased in cortex and epidermis due to the NO treatment. The PttTrHb localization that was found may reveal the involvement of PttTrHb in the NO metabolism.

## Supporting Information

Figure S1 **Specificity of the affinity purified PttTrHb antibody with Western blot of hybrid aspen leaf and stem extracts (250 Jg of protein).** 1, PageRuler Prestained Protein Ladder (Fermentas). 2, Proteins extracted from control leaves. 3, Proteins extracted from 5 h SNP-treated leaves. 4, Recombinant PttTrHb proteins expressed in *E*. *coli*. 5, Proteins extracted from 5 h SNP-treated stem. PttTrHb is localized at 19.2 kDa (arrow head). Molecular masses of marker proteins are represented in kDa. In leaves from control (Fig. S1A, B) or 5 h SNP-treated (Fig. S1A) plants, the level of PttTrHb was too low to be detected by Western blot hybridization. No background was observed even if the quantity of proteins used for the electrophoresis was high (250 Jg), confirming the specificity of the antibody.
(PDF)
