# Supplementary material for: Evolution, Three-Dimensional Model and Localization of Truncated Hemoglobin PttTrHb of Hybrid Aspen
Source: PLoS One. 2014 Feb 10;9(2):e88573. doi: 10.1371/journal.pone.0088573 (PMC3919811; doi:10.1371/journal.pone.0088573)
Supplement: Figure S1 — Specificity of the affinity purified PttTrHb antibody with Western blot of hybrid aspen leaf and stem extracts (250 Jg of protein). 1, PageRuler Prestained Protein Ladder (Fermentas). 2, Proteins extracted from control leaves. 3, Proteins extracted from 5 h SNP-treated leaves. 4, Recombinant PttTrHb proteins expressed in E. coli. 5, Proteins extracted from 5 h SNP-treated stem. PttTrHb is localized at 19.2 kDa (arrow head). Molecular masses of marker proteins are represented in kDa. In leaves from control (Fig. S1A, B) or 5 h SNP-treated (Fig. S1A) plants, the level of PttTrHb was too low to be detected by Western blot hybridization. No background was observed even if the quantity of proteins used for the electrophoresis was high (250 Jg), confirming the specificity of the antibody. (PDF) [file pone.0088573.s001.pdf]

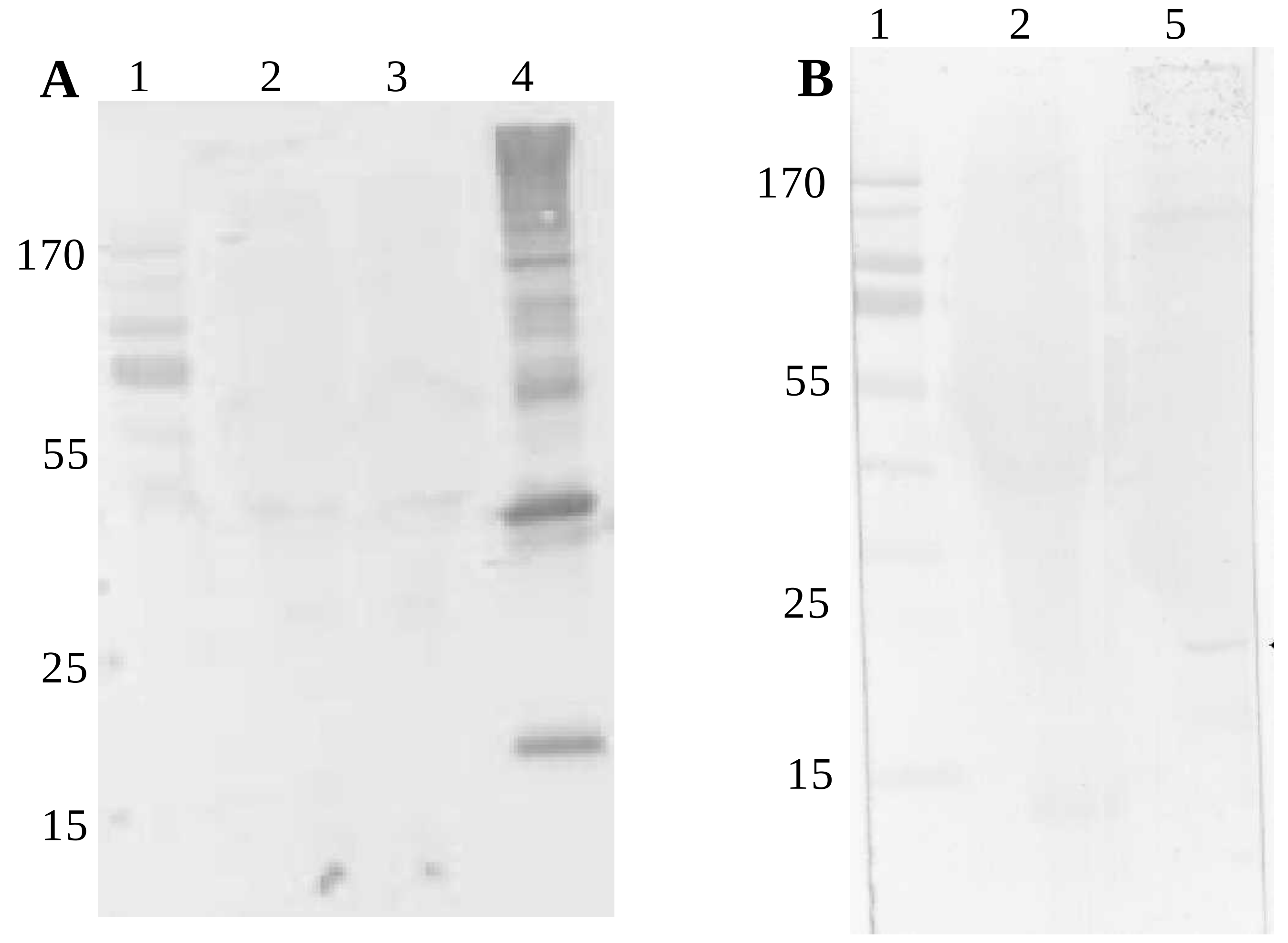

Fig. S1: Specificity of the affinity purified PttTrHb antibody with Western blot of hybrid aspen leaf and stem extracts (250 Jg of protein).

1, PageRuleri Prestained Protein Ladder (Fermentas). 2, Proteins extracted from control leaves. 3, Proteins extracted from 5 h SNP-treated leaves. 4, Recombinant PttTrHb proteins expressed in *E. coli*. 5, Proteins extracted from 5 h SNP-treated stem. PttTrHb is localized at 19.2 kDa (arrow head). Molecular masses of marker proteins are represented in kDa.

In leaves from control (Fig. 1A, B) or 5 h SNP-treated (Fig. 1A) plants, the level of PttTrHb was too low to be detected by Western blot hybridization. No background was observed even if the quantity of proteins used for the electrophoresis was high (250 Jg), confirming the specificity of the antibody.
